# A Novel Mouse Model for Stable Engraftment of a Human Immune System and Human Hepatocytes

**DOI:** 10.1371/journal.pone.0119820

**Published:** 2015-03-17

**Authors:** Helene Strick-Marchand, Mathilde Dusséaux, Sylvie Darche, Nicholas D. Huntington, Nicolas Legrand, Guillemette Masse-Ranson, Erwan Corcuff, James Ahodantin, Kees Weijer, Hergen Spits, Dina Kremsdorf, James P. Di Santo

**Affiliations:** 1 Innate Immunity Unit, Department of Immunology, Institut Pasteur, Paris, France; 2 Institut National de la Santé et de la Recherche Médicale (INSERM) U668, Paris, France; 3 Academic Medical Center at the University of Amsterdam, Amsterdam, The Netherlands; 4 Institut National de la Santé et de la Recherche Médicale (INSERM) U845, Faculté de Médecine Paris Descartes, Paris, France; Institut national de la santé et de la recherche médicale—Institut Cochin, FRANCE

## Abstract

Hepatic infections by hepatitis B virus (HBV), hepatitis C virus (HCV) and *Plasmodium* parasites leading to acute or chronic diseases constitute a global health challenge. The species tropism of these hepatotropic pathogens is restricted to chimpanzees and humans, thus model systems to study their pathological mechanisms are severely limited. Although these pathogens infect hepatocytes, disease pathology is intimately related to the degree and quality of the immune response. As a first step to decipher the immune response to infected hepatocytes, we developed an animal model harboring both a human immune system (HIS) and human hepatocytes (HUHEP) in BALB/c Rag2^-/-^ IL-2Rγc^-/-^ NOD.*sirpa* uPA^tg/tg^ mice. The extent and kinetics of human hepatocyte engraftment were similar between HUHEP and HIS-HUHEP mice. Transplanted human hepatocytes were polarized and mature *in vivo*, resulting in 20–50% liver chimerism in these models. Human myeloid and lymphoid cell lineages developed at similar frequencies in HIS and HIS-HUHEP mice, and splenic and hepatic compartments were humanized with mature B cells, NK cells and naïve T cells, as well as monocytes and dendritic cells. Taken together, these results demonstrate that HIS-HUHEP mice can be stably (> 5 months) and robustly engrafted with a humanized immune system and chimeric human liver. This novel HIS-HUHEP model provides a platform to investigate human immune responses against hepatotropic pathogens and to test novel drug strategies or vaccine candidates.

## Introduction

Infectious diseases that target the liver, including *Plasmodium* parasites and hepatitis B and C viruses (HBV, HCV), affect over 600 million people worldwide. The restricted species tropism of these hepatotropic pathogens has severely limited their study *in vivo* to non-human primates and transgenic or humanized mouse models. Studies in chimpanzees have been key to deciphering the role of the immune response in pathogen clearance and in testing vaccine candidates or treatment strategies; however the high cost, small cohort sizes and ethical concerns have limited their use [1,2]. Transgenic mouse models for HBV and HCV have been useful to investigate viral entry and the molecular mechanisms of viral replication [3–5], although the lack of viral spread and differences between mouse and human immune systems restricts their applications [6,7].

Humanized mice bearing chimeric human livers represent an important tool to study human hepatotropic infections [8,9]. Liver toxicity generated by hepatocyte-specific expression of the urokinase plasminogen activator (uPA) transgene creates a ‘niche’ for growth of adoptively transferred hepatocytes [10,11]. Human hepatocytes can repopulate the liver of immunodeficient uPA transgenic mice [12], the resultant human hepatocyte (HUHEP) engrafted mice are permissive for HBV and HCV infections and establish the complete viral life cycle *in vivo* [9,12,13]. HUHEP mice established from immunodeficient animals lacking the fumaryl acetoacetate hydrolase (FAH) gene are also susceptible to HBV and HCV infections [14,15]. However, these HUHEP models cannot be used to monitor anti-viral immune responses, as recipient mice are immunodeficient.

Humanized mice harboring a human immune system (HIS mice) have shown promise for dissecting human immune responses *in vivo* [7,16–18]. HIS mice can be established by injecting human hematopoietic stem cells (HSC) into immunodeficient mice, generating multi-lineage engraftment and robust immune system repopulation [19,20]. Such HIS mice are susceptible to human lymphotropic infections (including HIV, EBV, HTLV-1) and can serve as a platform to study human anti-viral immune responses, as well as prophylactic and therapeutic treatments for these infections [18].

Recently, efforts to obtain immunocompetent humanized mouse models with chimeric human livers have been described in which syngeneic human hepatoblasts and HSC were injected, however only low levels of human hepatocyte engraftment were observed [21,22]. Alternatively, co-injection of HLA-mismatched HSC and adult human hepatocytes could enable high levels of liver chimerism, although the development of the humanized immune system in this context was not fully characterized or compared to existing HIS models [22,23].

In this report, we describe a novel mouse model for co-engraftment of human immune cells and human hepatocytes (HIS-HUHEP mice) generated in BALB/c Rag2^-/-^ IL-2Rγc^-/-^ NOD.*sirpa* recipients that harbor the urokinase plasminogen activator (uPA) transgene. HIS-HUHEP mice demonstrate high levels of liver chimerism as well as robust engraftment of human myeloid and lymphoid cell subsets that are maintained for at least 5 months. We propose that this novel HIS-HUHEP mouse model will find utility as a technology platform to decipher the human immune response to hepatotropic pathogens *in vivo* [24].

## Materials and Methods

### Mouse models and human cell engraftment

BALB/c Rag2^-/-^IL-2Rγc^-/-^ NOD.*sirpa* (BRGS) congenic mice [17] were bred with BALB/c Rag2^-/-^IL-2Rγc^-/-^uPA^tg/wt^ mice [25], to establish a BALB/c Rag2^-/-^IL-2Rγc^-/-^ NOD.*sirpa* uPA^tg/wt^ (BRGS-uPA) colony. To generate HIS and HIS-HUHEP mice, newborn (<1w old) BRGS and BRGS-uPA^tg/tg^ mice were sublethally irradiated (2,5Gy) and injected intrahepatically with ~2x10^5^ CD34^+^ or ~7x10^4^ sorted CD34^+^CD38^-^ human fetal liver cells, according to previously described protocols [26] (Advanced Bioscience Resources). HUHEP and HIS-HUHEP mice were obtained by injecting 7x10^5^ freshly thawed human hepatocytes (Biopredic or BD Biosciences) into the spleen of 4–8 week old BRGS-uPA^tg/tg^ naïve mice (HUHEP), or mice previously injected with CD34^+^ cells (HIS-HUHEP) following previously described protocols [27].

### Ethics statement

Animals were housed in isolators under pathogen-free conditions with humane care and anesthesia was performed using Ketamine and Xylazine to minimize suffering. Experiments were approved by an ethical committee at the Institut Pasteur (Reference # 2007–006) and validated by the French Ministry of Education and Research (Reference # 02162.01).

### ELISA and flow cytometric analysis

Human albumin (cat# E80–129 Bethyl Laboratories), human IgM (cat# 109–035–043 Jackson ImmunoResearch), and human IgG (cat# 109–035–008 Jackson ImmunoResearch) levels were quantified in the plasma by ELISA according to the manufacturer’s instructions and previously described protocols [16]. Mononuclear cells from blood, spleen, bone marrow, thymus and lymph nodes were isolated as previously described [16]. Liver leukocytes were isolated after eliminating circulating cells by perfusing the liver with PBS *in situ* through the vena cava as previously described [28]. To test the functionality of T cells, splenocytes were cultured in IMDM with 10% fetal calf serum, 10 IU/ml hIL-2 (Chiron), 100 ng/ml hIL-7 (Miltenyi), 4x10^5^ Dynabeads CD3/CD28 (Life technologies) for 9 days then stimulated for 4 hours with 50 ng/ml PMA and 1 microg/ml ionomycin for 4 hours prior to intracellular cytokine staining using the BD cytofix/ cytoperm method (BD biosciences). Flow cytometry was performed with directly conjugated antibodies according to standard techniques using a Fortessa and LSRII flow cytometers (Becton Dickinson). LIVE/DEAD Fixable Aqua Dead Cell Stain Kit (Invitrogen) and a 405 nm excitation were used to exclude dead cells. The list of anti-human antibodies used in flow cytometry is detailed in S1 Table. Analysis was performed with Flowjo Version 8.8 (TreeStar).

### Immunohistological analysis

Cryostat sections of liver lobes were prepared for immunofluorescence staining as previously described [28]. The following anti-human antibodies were used: albumin (cat# A0001 Dako) and (cat# A80–129A Bethyl), CD45 (clone 2D1 BD Biosciences), Cyp2C9 (cat#AHP617Z AbD Serotec), Cyp3A4 (cat# AHP622Z AbD Serotec). The anti-E-cadherin (clone NCH-38, Dako), and anti-Occludin-1 (clone OC-3F10 Zymed) antibodies detect human and mouse antigens. Secondary antibodies were coupled to Alexa Fluor 488, Alexa Fluor 555, or Alexa Fluor 647 (Molecular Probes), slides were mounted with SlowFade Gold antifade reagent with DAPI (Invitrogen).

### Statistical analysis

Statistical significance of the data was tested with two-tailed unpaired Student t test analysis and correlations were analyzed with Pearson’s Χ^2^ test using GraphPad Prism version 6 for Mac (GraphPad Software).

## Results and Discussion

In order to establish an animal model in which human immune responses against human hepatotropic pathogens could be studied *in vivo*, we generated a novel recipient mouse strain that could be co-engrafted with human HSC and human hepatocytes. We therefore interbred immunodeficient BALB/c Rag2^-/-^IL-2Rγc^-/-^ NOD.*sirpa* (BRGS) congenic mice that have been used to create HIS mice, with BALB/c Rag2^-/-^IL-2Rγc^-/-^ uPA^tg^ mice that have been used to create HUHEP mice. The resultant BALB/c Rag2^-/-^IL-2Rγc^-/-^ NOD.*sirpa* uPA^tg^ (BRGS-uPA) mice were then intercrossed to obtain BRGS-uPA^tg/tg^ animals. Previous studies demonstrated that reduced fertility in uPA^tg/tg^ animals could be alleviated following engraftment of mouse hepatocytes, thus we also engrafted the BRGS-uPA^tg/tg^ breeders to improve their performance. Accordingly, we did not observe neonatal lethality and routinely obtained normal litter sizes (n = 5–8) using this novel BRGS-uPA^tg/tg^ strain.

**Fig 1:**
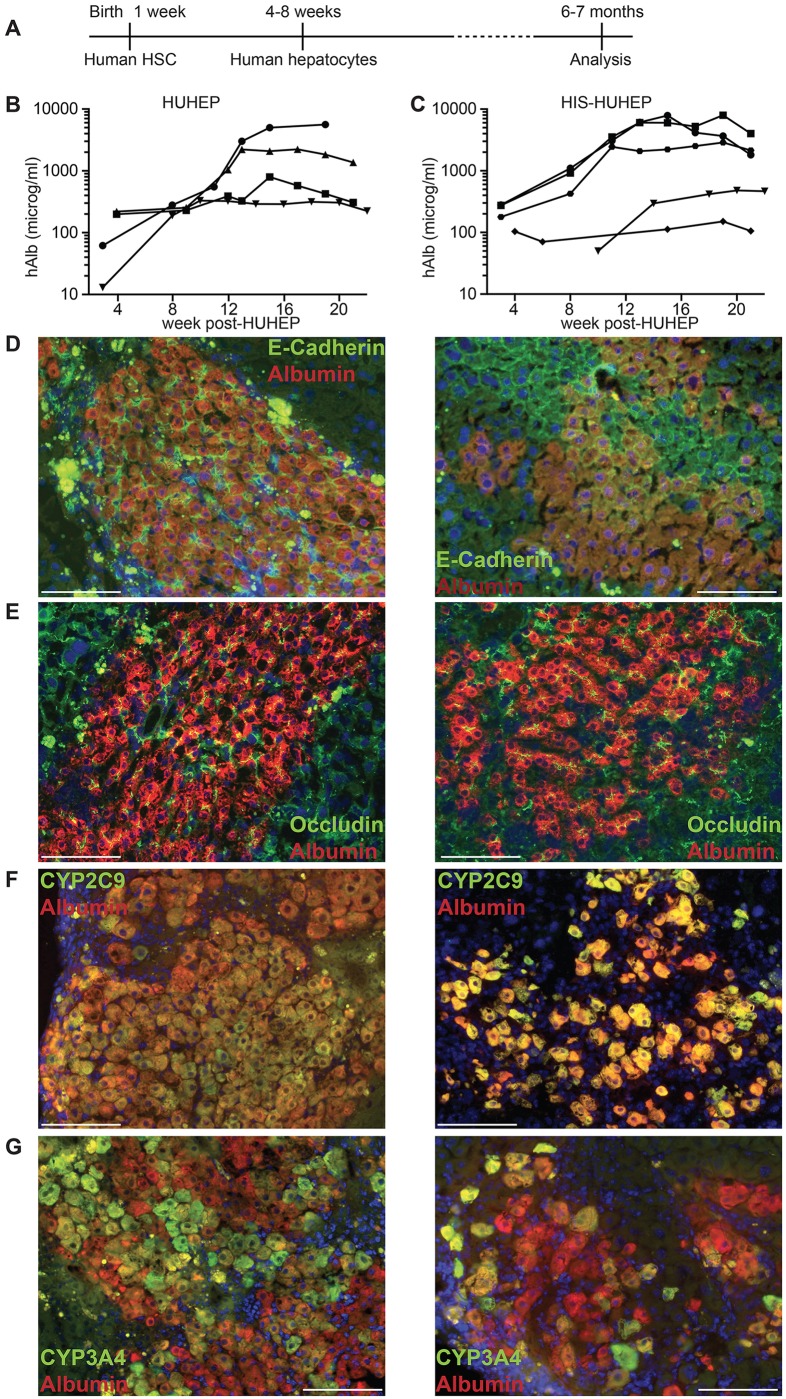
Experimental timeframe and analysis of human hepatocyte engraftment in HUHEP and HIS-HUHEP mice. (A) Time course of the experiment indicating the age of the mice at each step. Human albumin (hAlb) plasma levels were analyzed longitudinally in HUHEP (n = 4) (B) and HIS-HUHEP (n = 5) (C) mice. (D, E, F and G) Immunofluorescence analysis of HUHEP (left) and HIS-HUHEP (right) liver sections for anti-hAlb (red stain) co-stained with either (D) anti-E-Cadherin (green stain), or (E) anti-Occludin (green stain), or (F) anti-human Cyp2C9 (green stain), or (G) anti-human Cyp3A4 (green stain) expression. Nuclei are stained with DAPI (blue). Scale bar represents 100 microm.

BRGS-uPA^tg/tg^ mice were engrafted in a two-step process to obtain HIS-HUHEP mice: irradiated newborn pups (< 1 week old) were injected intrahepatic ally with human HSC, and at 4–8 weeks they were implanted with human adult hepatocytes (Fig. 1A). The experimental protocol was designed in accordance with previous studies showing higher levels of engraftment and more robust multi-lineage differentiation following human HSC injection into newborns compared to adults. Furthermore, we chose to inject human adult hepatocytes since they were previously shown to engraft and differentiate *in vivo* more efficiently than human fetal liver progenitor cells, thus leading to higher levels of liver chimerism [29]. The overall survival rate at the end of this procedure was 70%.

### Liver chimerism in HUHEP versus HIS-HUHEP mice

We first compared human hepatocyte engraftment efficiency in HUHEP versus HIS-HUHEP mice. Liver chimerism by human hepatocytes was followed longitudinally (over 5 months) by monitoring serum human albumin (hAlb) levels (Fig. 1B and C). In both HUHEP and HIS-HUHEP mice, high hAlb levels (100–10,000 microg/ml) were routinely obtained, reflecting 20–50% human hepatocyte repopulation of the mouse liver [30]. As previously described for the HUHEP model, hepatocyte engraftment in the HIS-HUHEP mice steadily increased with time and was stable for at least 5 months post-hepatocyte graft (Fig. 1B and C). No significant differences in the kinetics and the overall level of hepatocyte engraftment were observed in HUHEP versus HIS-HUHEP mice.

In the liver, hepatocytes are polarized along the sinusoidal (basolateral) and canalicular (apical) domains where the adherens-junction protein E-cadherin and the tight-junction protein Occludin-1 are concentrated respectively [31,32]. In both HUHEP and HIS-HUHEP livers, the human hepatocytes properly localized E-cadherin to the basolateral pole (Fig. 1D). Occludin-1 was expressed at the tight junctions of the apical pole and revealed the bile canalicular channels running between adjacent hepatocytes (Fig. 1E). To determine whether engrafted human hepatocytes were functionally mature, their expression of two major cytochrome (CYP) P450 (CYP2C9 and CYP3A4) xenobiotic and drug-metabolizing phase I enzymes were assessed. In both models all human hepatocytes expressed CYP2C9, whereas a fraction of cells expressed CYP3A4 (Fig. 1F and G). Thus, in HUHEP and HIS-HUHEP livers, human hepatocytes were fully integrated into the mouse parenchyma, with normal polarization, differentiation, and the engraftment was robust and stable over time.

### Humanization of the immune system in HIS versus HIS-HUHEP mice

**Fig 2:**
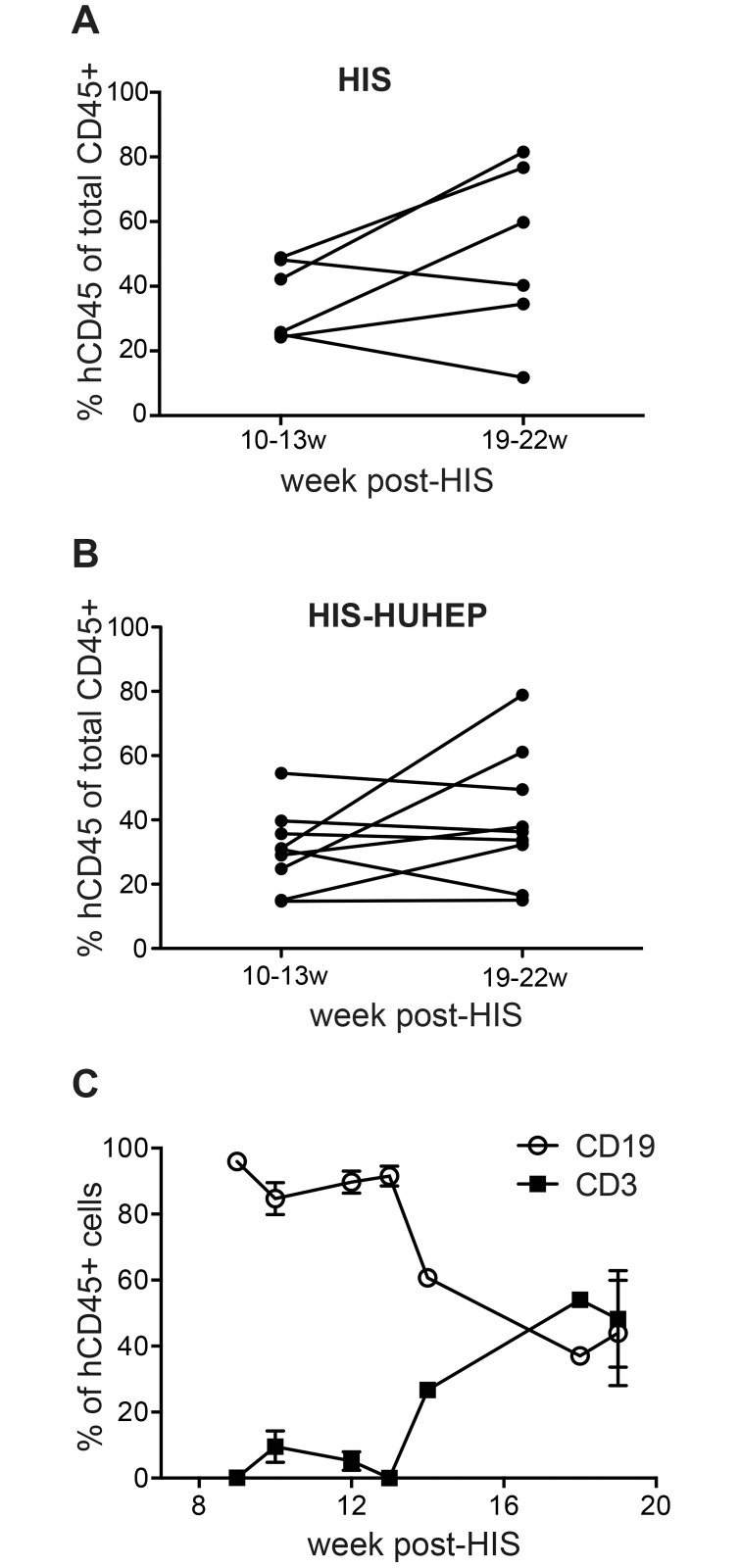
Evaluation of circulating human hematopoietic cells in HIS and HIS-HUHEP mice. Frequency of human leukocytes (hCD45^+^) measured by FACS in the blood of (a) HIS and (B) HIS-HUHEP mice at early (10–13w) and late (19–22w) time points post-hematopoietic stem cell engraftment. Each dot represents a mouse. (C) Relative proportions of T (CD3^+^) and B (CD19^+^) cells within hCD45^+^ cells from the blood of HIS-HUHEP mice sampled longitudinally (n = 10 mice).

To characterize the development of the human immune system over time, we compared circulating human hematopoietic cells in HIS and HIS-HUHEP mice at early (10–13 weeks) and late (19–22 weeks) time points after HSC injection (Figs. 2a and 2B). The frequency of hCD45^+^ cells in the blood was similar at both time points for each model, as well as between the different models at each time point. This data suggests that human HSC develop at a similar rate in the HIS and HIS-HUHEP models. Longitudinal analysis of T and B lymphocyte migration in the blood of HIS-HUHEP mice showed that circulating B lymphocytes appeared first, whereas peripheral T cell accumulation occurred later (Fig. 2C), as has been previously described [33].

**Fig 3:**
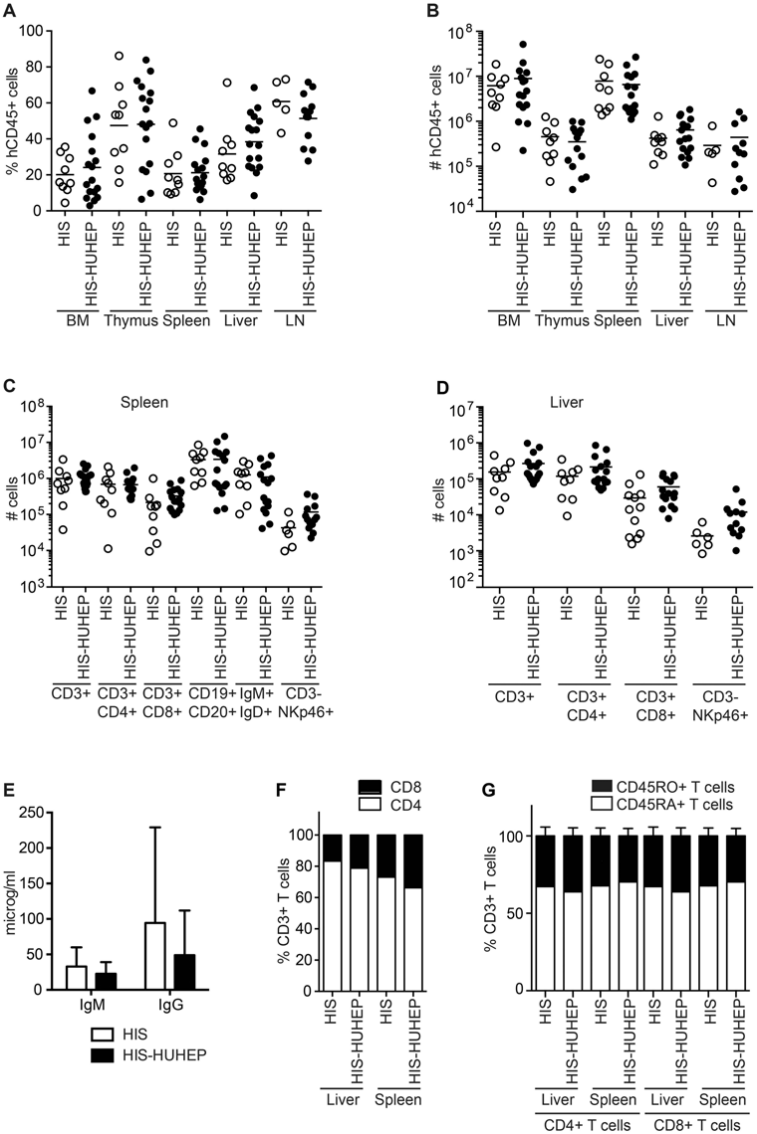
Analysis of human immune cells in HIS and HIS-HUHEP mice. The (A) percentage and (B) total number of hCD45^+^ cells in the bone marrow (BM), thymus, spleen, liver and lymph nodes (LN) in HIS and HIS-HUHEP mice were determined. Absolute numbers of lymphocyte subsets in the (C) spleen and (D) liver of HIS and HIS-HUHEP mice. (E) Total human IgM and IgG concentrations were measured in the plasma of humanized mice. Error bars show standard deviation. (F) Relative proportions of CD4^+^ T_H_ and CD8^+^ T_C_ cell subsets within total CD3^+^ T cells in the liver and spleen of HIS and HIS-HUHEP mice. (G) Relative percentages of naive (CD45RA^+^ white bar) or memory (CD45RO^+^ black bar) CD3^+^ T cells in the liver and spleen of HIS and HIS-HUHEP mice. No statistically significant differences were observed between the HIS and HIS-HUHEP groups (unpaired t tests).

The tissue distribution of hematopoietic cell subsets was compared in the singly and doubly humanized mouse models at 24–29 weeks post injection. Primary (bone marrow (BM), thymus) and secondary lymphoid organs (spleen, mesenteric lymph nodes (mLN)) and non-lymphoid tissues (liver) were reconstituted with hCD45^+^ cells at comparable frequencies and the total hCD45^+^ cellularity in each organ was similar in the HIS and HIS-HUHEP models (Figs. 3A and 3B). We observed comparable levels of reconstitution of human CD4^+^ T helper (T_H_), CD8^+^ T cytotoxic (T_C_), and NK (CD3^-^ NKp46^+^) lymphocytes within the splenic and hepatic compartments in these two models (Figs. 3C and 3D). In the spleen, both models had similar numbers of B cells (CD19^+^CD20^+^) and of transitional or mature B cells (IgM^+^ IgD^+^) (Fig. 3C). These B lymphocytes were functional and produced human IgM and IgG antibodies measured at comparable levels in the plasma of HIS and HIS-HUHEP mice (Fig. 3E). Splenic and hepatic CD3^+^ T cells demonstrated a biased CD4/CD8 ratio (with a majority of CD4^+^ T_H_ cells) in both models (Fig. 3F) as has been previously described in other HIS models [34]. Importantly, the majority of splenic and hepatic T_H_ and T_C_ cells displayed a naïve phenotype (CD45RA^+^CD45RO^-^) in HIS and HIS-HUHEP mice (Fig. 3G). Human T cells from HIS and HIS-HUHEP mice were functional as splenocyte-derived CD4^+^ T_H_ and CD8^+^ T_C_ cells responded to *in vitro* stimulation with PMA+ionomycin by producing TNFα and IFNγ (in HIS and HIS-HUHEP respectively 21–31% and 43–70% of TNFα^+^ IFNγ^+^ CD4^+^ cells, in HIS and HIS-HUHEP respectively 28–58% and 26–30% of TNFα^+^ IFNγ^+^ CD8^+^ cells, n = 3 in each group).

**Fig 4:**
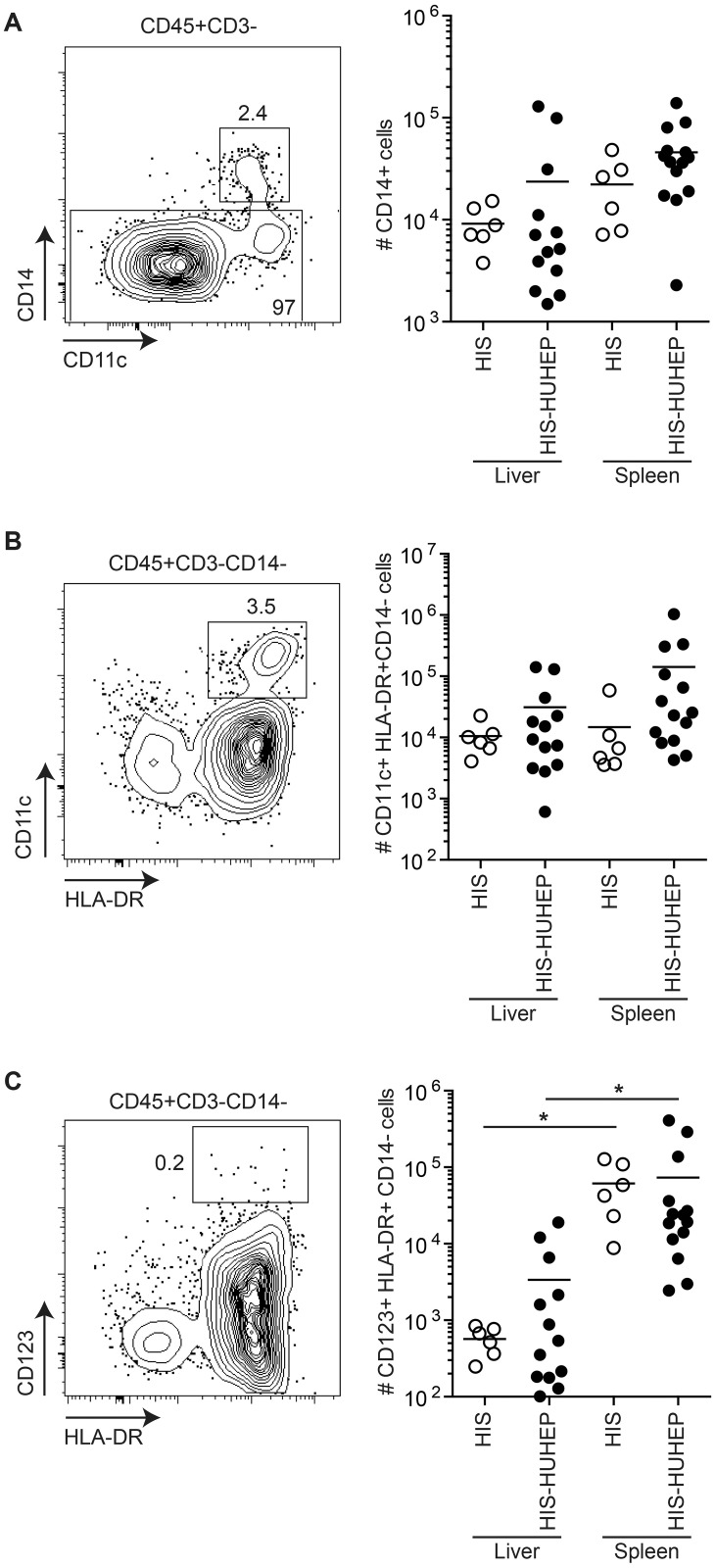
Characterization of human myeloid cells in HIS and HIS-HUHEP mice. Representative FACS contour plots of liver-isolated cells from HIS-HUHEP mice are shown for each cell subset with the parental gating strategy indicated above the plot. Total numbers of human (A) monocytes (hCD45^+^ CD3^-^ CD14^+^), (B) myeloid dendritic cells (hCD45^+^ CD3^-^ CD14^-^ CD11c^+^ HLA-DR^+^), and (C) plasmacytoid dendritic cells (hCD45^+^ CD3^-^ CD14^-^ CD123^+^ HLA-DR^+^) were determined in the liver and spleen of HIS and HIS-HUHEP mice. * p< 0,05 unpaired two-tailed t test.

Myeloid cells play a central role in antigen presentation and initiating immune responses following infections, thus we analyzed the human myeloid cell subsets in the liver and the spleen of these mice. Human monocytes (CD14^+^) and myeloid dendritic cells (mDC CD14^-^CD11c^+^HLA-DR^+^) were present in similar numbers in the liver and spleen of HIS and HIS-HUHEP mice (Figs. 4A and 4B). Human plasmacytoid dendritic cells (pDC CD14^-^CD123^+^HLA-DR^+^) were more numerous in the spleen than in the liver of HIS and HIS-HUHEP mice (Fig. 4C). Thus singly and doubly humanized mouse models develop myeloid cell subsets similarly to previously described HIS models [35,36].

**Fig 5:**
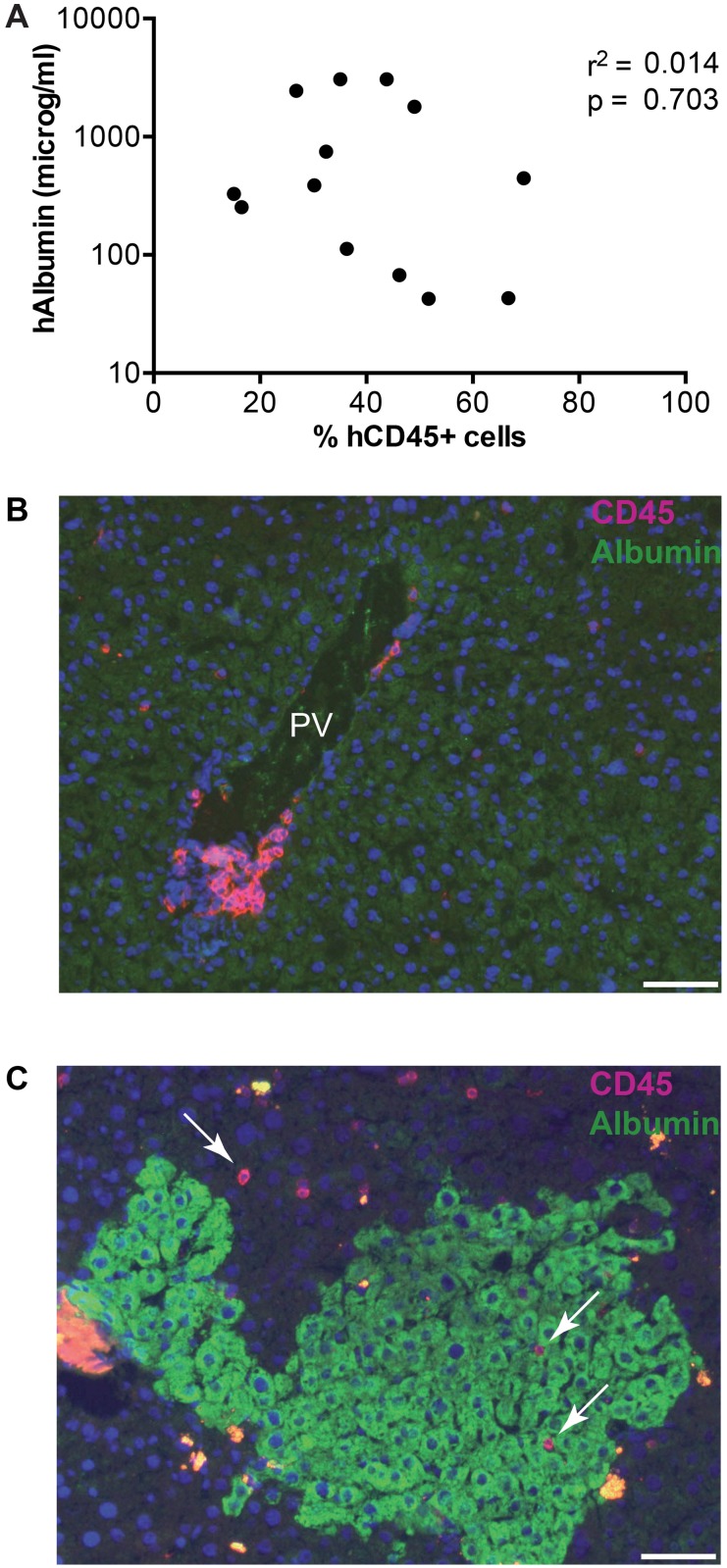
Evaluation of human immune cells and human hepatocytes in HIS-HUHEP mice. (a) Correlation analysis of the percentage of hCD45^+^ cells and hAlbumin in the blood of HIS-HUHEP mice sampled at 18–21w post-HIS and 10–16w post-HUHEP reconstitution. Each dot represents a mouse; correlation was analyzed with Pearson’s Χ^2^ test. (B and C) Immunofluorescence analysis of intrahepatic leukocyte infiltration in (B) HIS and (C) HIS-HUHEP livers showing hCD45^+^ cells (purple) and hAlbumin^+^ cells (green). PV: portal vein, white arrows indicate hCD45^+^ cells. Nuclei are stained with DAPI (blue). Scale bar represents 100 microm.

Since the HSC and hepatocytes used to create HIS-HUHEP mice are not derived from the same donor, we investigated whether any graft (HSC) vs. graft (hepatocyte) reaction might be occurring. This seemed unlikely for several reasons. First, hAlb levels were stable for at least 5 months in HIS-HUHEP mice (Fig. 1C) demonstrating that the hepatocyte graft is stable in the presence of an apparently allogeneic immune system. Second, numbers of splenic and hepatic hCD45^+^ cells (including CD3^+^ T cells) were similar in HIS and HIS-HUHEP mice (Figs. 3B, 3C, and 3D), and retained a naïve phenotype (Fig. 3G) consistent with an absence of immune expansion or activation. Third, reconstitution of the immune system (%hCD45^+^ cells in the blood) was not correlated to human liver chimerism (hAlb in the plasma) in a cohort of HIS-HUHEP mice (r^2^ = 0.014 p = 0.703) (Fig. 5A). Finally, no inflammatory immune cell infiltration was detected near the human hepatocyte grafts in the HIS-HUHEP model: hCD45^+^ leukocytes were observed in the portal vein of HIS mice (Fig. 5B) and infrequently in the liver parenchyma of HIS-HUHEP mice (Fig. 5C).

Taken together, these results demonstrate robust and durable engraftment of a human immune system and human hepatocytes in the HIS-HUHEP model, with similar kinetics and efficiency of repopulation as the singly humanized HIS and HUHEP models.

## Conclusions

We have developed a novel humanized mouse model that can be stably co-engrafted with a human hemato-lymphoid system and human hepatocytes for at least 6–7 months. This HIS-HUHEP model should provide a means to study the ‘cross-talk’ *in situ* between the liver and the immune system during hepatotropic infections. Although this model must be validated by challenging the mice with hepatotropic pathogens, we first performed an in depth analysis and comparison of the dually and singly humanized models in order to fully characterize the stability and extent of co-engrafted human cells.

In the liver of HIS-HUHEP mice, human hepatocytes extensively engraft generating hAlb levels of 100–10,000 microg/ml in the plasma that correspond to approximately 20–50% replacement of the mouse liver by human cells. The engraftment of human hepatocytes in our HIS-HUHEP model is similar to what has been observed in the uPA-NOG (uPA-NOD/*scid*/IL2Rg^null^) and FRGN (Fah^-/-^Rag2^-/-^IL2Rγ^-/-^ NOD) hosts, and greater than in the AFC8 (albumin promoter FKBP-Caspase 8) host [21–23]. This high level of human hepatocyte chimerism should render HIS-HUHEP mice susceptible to species-restricted hepatotropic pathogens (HBV, HCV or *P*. *falciparum*) [9,37]. Excessive levels (≥90%) of human repopulation in the liver can be deleterious to the host, since murine metabolic functions must be maintained and high levels of human complement are toxic in the mouse [38]. Human hepatocytes engrafted in humanized chimeric mice were apically and basally polarized, with tight junctions delimitating bile canalicular channels forming between adjacent cells in the host mouse liver. The detoxifying activities of hepatic phase I and phase II enzymes are species specific, thus HUHEP mice have been very useful for drug metabolism and pharmacokinetic (DMPK) studies [39]. The presence of human Cyp2C9 and Cyp3A4 in human hepatocytes engrafted in HIS-HUHEP mice suggests that this model could also be valuable for toxicology studies. It will be interesting to determine whether the presence of a humanized immune system can modulate DMPK responses during viral infections. Together, these results indicate that the hepatic phenotype of the HUHEP and HIS-HUHEP models are comparable, with proper maintenance of liver architecture and hepatic functions.

In both HIS and HIS-HUHEP mice the primary, secondary, and non-lymphoid (liver) tissues were reconstituted with human myeloid and lymphoid cells. The engraftment kinetics was similar in the two models and comparable to previous reports [33]. Efficient immune responses following infection are mediated by the rapid recruitment of myeloid cells to the site of infection, which induces activation of the cellular response by NK and T lymphocytes, followed by the humoral response of B cells. In both the HIS and HIS-HUHEP mice, monocytes and dendritic cells developed with low frequencies that were similar to NOD/SCID and NOD/SCID/IL2rγ^null^ (NSG) recipients [35,36]. Interestingly, most T cells were naïve (CD45RA^+^) in both the liver and spleen of HIS and HIS-HUHEP mice (Fig. 3G) and therefore should be capable of normally responding to immune stimulation. Mature B lymphocytes developed in the HIS and HIS-HUHEP models, producing levels of human Igs that are comparable to those reported in previous BRG and BRGS HIS mice [17,33], and roughly 10 to 100-fold higher than observed in NSG-based models [34,40].

Although the human HSC and human hepatocytes were not HLA matched, we did not observe any graft (immune) versus graft (hepatocyte) (GvG) response in HIS-HUHEP mice. As hepatocyte transplantation is performed only 4–8 weeks post-HSC injection, that is well before mature T cells have appeared in peripheral organs (Fig. 2C) and [33], we speculate that hepatocyte-derived alloantigens are available and presented to developing thymocytes rendering them tolerant. Another explanation for the lack of GvG response lies in the tolerogenic nature of the liver microenvironment, where intra-hepatic antigen-presenting cells can skew immune responses to an immunoregulatory phenotype, thereby shielding hepatocytes from immune attack. The ability to create HIS-HUHEP mice using the widely available chimeric liver model (uPA mice) and cells from un-related donors should greatly facilitate the generation and accessibility of this model.

The immune response plays an essential role in mediating viral eradication or persistence during heptotropic infections. The susceptibility of singly humanized HIS or HUHEP mice to respectively lymphotropic (HIV, EBV, Dengue) and hepatotropic (HBV, HCV, P. falciparum) infections, suggests that this novel doubly humanized model may be a suitable platform to study the immune response to mono-infections as well as co-infections. Eventually, HIS-based models could be applied to testing novel therapeutic strategies or vaccine candidates.

## Supporting Information

S1 Table Antibodies used for flow cytometry analysis. (PDF)
