# Supplementary material for: A Novel Mouse Model for Stable Engraftment of a Human Immune System and Human Hepatocytes
Source: PLoS One. 2015 Mar 17;10(3):e0119820. doi: 10.1371/journal.pone.0119820 (PMC4364106; doi:10.1371/journal.pone.0119820)
Supplement: S1 Table — (PDF) [file pone.0119820.s001.pdf]

Table S1.
Antibodies used for flow cytometry analysis.

Antigen	Reactivity	Clone	Supplier
CD45.2	Mouse	104	Biolegend
CD45	Human	5B1	Miltenyi
CD4	Human	VIT4	Miltenyi
CD8	Human	RPA-T8	BD Biosciences
CD3	Human	UCHT1	BD Biosciences
CD45RO	Human	UCHL1	Miltenyi
CD45RA	Human	T6D11	Miltenyi
HLA-DR	Human	L243	Ebioscience
CD19	Human	LT19	Miltenyi
CD20	Human	2H7	Biolegend
IgM	Human	G20-127	BD Biosciences
IgD	Human	IA6-2	BD Biosciences
CD14	Human	TUK4	Miltenyi
CD123	Human	6H6	Ebioscience
CD11c	Human	3.9	Ebioscience
NKp46	Human	9E2/NKp46	BD Biosciences
IFNγ	Human	45-15	Miltenyi
TNFα	Human	cA2	Miltenyi
